# Iron deficiency and thyroid dysfunction among sudanese women in first trimester of pregnancy: a cross-sectional study

**DOI:** 10.1186/s12902-023-01487-z

**Published:** 2023-10-13

**Authors:** Wisal Abbas, Abdelmageed Elmugabil, Hamdan Z. Hamdan, Duria A. Rayis, Ishag Adam

**Affiliations:** 1 Faculty of Medicine, Kordofan University, Elobeid, Sudan; 2 Faculty of Medicine, El Imam El Mahdi University, Kosti, Sudan; 3 Department of Basic Medical Sciences, Unaizah College of Medicine and Medical Sciences, Qassim University, Unaizah, 51911 Saudi Arabia; 4 Department of Biochemistry and Molecular Biology, Faculty of Medicine, Al-Neelain University, P.O. BOX 12702, Khartoum, Sudan; 5 Faculty of Medicine, University of Khartoum, Khartoum, Sudan; 6 Department of Obstetrics and Gynecology, Unaizah College of Medicine and Medical Sciences, Qassim University, Unaizah, Saudi Arabia

**Keywords:** Anemia, Iron Deficiency, Pregnancy, Thyroid, Thyroxine, Triiodothyronine

## Abstract

**Background:**

Pregnant women are more susceptible to iron deficiency (ID), and it can lead to several maternal and perinatal adverse effects. There are some published data on the effect of ID on thyroid function, but none of the studies were conducted in sub-Saharan African countries including Sudan. This study was conducted to investigate association between ID (ferritin < 15 µg/L) and thyroid functions [thyroid-stimulating hormone (TSH), free triiodothyronine (FT3), and free thyroxine (FT4)] among Sudanese women in the first trimester of pregnancy.

**Method:**

A cross-sectional study was conducted in Saad Abuelela Maternity Hospital, Sudan. Obstetric/sociodemographic characteristics were gathered through questionnaires. Hemoglobin, serum ferritin, TSH, FT3, and FT4 were measured in all pregnant women. Continuous variables were compared with either independent sample t-test if they were normally distributed, or with Mann–Whitney U- test if they were not-normally distributed. Spearman correlations were performed between the continuous variables.

**Results:**

In total, 127 pregnant women with mean [standard deviation (SD)] age of 27.0 (5.5) years and gestational age of 10.5 (3.0) weeks, respectively, were enrolled in this study. Forty-seven (37.0%) of these 127 women had ID. While the median [interquartile range (IQR)] of the parity, TSH, and FT3 were not different between women with ID and women without ID, the median (IQR) of FT4 was significantly lower in women with ID compared with women without ID [1.020 (0.910‒1.120) vs. 1.095 (0.990‒1.217) pmol, P = 0.014]. Serum ferritin was inversely correlated with FT3, (r = -0.225, P = 0.011). There was no significant correlation between serum ferritin, TSH, and FT4.

**Conclusions:**

Iron deficiency was common during the first trimester of pregnancy and was associated with thyroid dysfunctions. Therefore, ID should be evaluated to avoid thyroid dysfunction.

## Background

Iron deficiency (ID) is a major health problem that affects about one-fifth of the world’s population [1]. It continues to be a common and widespread problem during pregnancy in developing countries because of maternal malnutrition and the increased demand for iron [1, 2]. There is an increased need for iron during pregnancy due to the excessive expansion of maternal red blood cell mass and growth of both the fetus and placenta; therefore, pregnant women are vulnerable to ID [1, 3, 4]. ID can also lead to several adverse effects such as low birth weight, preterm birth, intrauterine growth restriction, and maternal mortality [5].

Thyroid dysfunction during pregnancy can lead to several maternal and perinatal adverse outcomes such as preeclampsia, preterm delivery, placental abruption [2, 6], miscarriage, gestational diabetes (GDM), low birth weight, and neonatal death [2]. Moreover, both ID and thyroid dysfunction can lead to several adverse maternal as well as perinatal complications, such as placental abruption, hypertension, miscarriage, and GDM [2, 3, 7]. Several previous studies have shown that ID during pregnancy was associated with thyroid dysfunction in the form of higher “serum thyroid-stimulating hormone (TSH), and lower free triiodothyronine (FT3) and lower free thyroxine (FT4) concentrations, and lower total thyroxine (TT4) level” [2–4, 8–11]. However, a recent study conducted in India found no such association between ID and hypothyroidism [12]. It worth mentioning, all these studies_ including the Indian study_, were conducted outside sub-Saharan Africa, where there is high prevalence of anemia, and the cause of anemia could be different from that of other countries. Therefore, there is a need to conduct research on ID and thyroid function in sub-Saharan African countries and in Sudan as one example of these countries. A recent meta-analysis has shown that anemia and ID are big health problems among pregnant Sudanese women [13]. The aim of this study was to evaluate the association between ID and thyroid function (TSH, FT3, and FT4) in Sudanese pregnant women in the first trimester of pregnancy. The findings of the present study are expected to yield data on thyroid dysfunction as well as ID during early pregnancy. This will help care providers and health planners with evidence-based data and guide proper interventions.

## Methods

### Study design

This study was a prospective hospital-based cross-sectional study conducted between January and April 2021 in Saad Abuelela Maternity Hospital (out-patient), which is governed by the Faculty of Medicine, University of Khartoum. The hospital is located in Khartoum city, the capital of Sudan.

Systematic random sampling was used to select the pregnant women in their early pregnancy who fulfilled the inclusion criteria. To perform this sampling technique, we use hospital records to make initial estimates. According to the hospital records, there were 400 pregnant women in their early pregnancy attended the antenatal care hospital over the four-month which preceded the study. The sampling interval ( ≈ 3) was reached by dividing the expected number of pregnant women (400) in the period of study by the required sample size which was 127 women (400/127 ≈ 3). Then the pregnant women were therefore interviewed at three intervals until the required sample size (127) was reached.

### Study populations

#### Inclusion criteria

Pregnant Sudanese women, life, singleton pregnancy and in the first trimester of pregnancy (< 14 weeks of gestational age).

#### Exclusion criteria

Women with previous thyroid disorders, diabetes mellitus, hypertension, any other chronic disease, hemolytic disease, and those who declined to participate; women with multiple pregnancies were also excluded from the study. Pregnant women were approach to participate in the study during the antenatal care visit. After signing an informed consent form, the participants completed a questionnaire on obstetric/sociodemographic and medical characteristics (age, data on previous pregnancies, and gestational age). The pregnancy itself and its duration were confirmed by ultrasonography. Body mass index (BMI) was computed from the height and weight, which were measured using the standard method.

### Methods of sampling and laboratory testing

Samples of blood were obtained from each participant. The maternal hemogram was measured for all women, using an automated hematology analyzer (Sysmex KX-21, Japan) as per manufacturer’s instructions. Five ml of venous blood was taken from each pregnant woman into plain serum bottles, allowed to clot, centrifuged to separate the serum, and stored at -20 °C until analyzed for measurements of serum ferritin and serum thyroid profile (FT3, FT4, and TSH) levels.

Radioimmunoassay gamma counter (Riostad, Germany) was used to measure maternal serum ferritin, using kits which were provided by Beijing Isotope Nuclear Electronic Co., Beijing, China. Iron deficiency was defined as serum ferritin levels < 15 µg/L.

Maternal serum thyroid (FT3, FT4, and TSH) levels were measured using the immunoassay analyzer AIA 360 (Tosoh Bioscience, San Francisco, CA, USA) in accordance with the manufacturer’s instructions. Samples were run twice and the mean value was computed as the final level.

### Sample size calculation

A sample size of 127 women was calculated using the formula to assess correlation:

$${\rm{N}}\,{\rm{ = }}\,{\left( {\left[ {{{\rm{Z}}_{\rm{\alpha }}}{\rm{ + }}\,{{\rm{Z}}_{\rm{\beta }}}} \right]{\rm{/C}}} \right)^{\rm{2}}}{\rm{ + }}\,{\rm{3}}$$

where, *N* = number of subjects required.

*Z*_α_ = the standard deviation for α.

Z_β_ = the standard deviation for β.

*C* = 0.5*ln ([1 + *r*]/[1 - *r*])

*r* = expected correlation coefficient.

The sample size of 127 women was calculated in order to detect a significant minimum level (r = 0.25) of correlation between serum ferritin and the levels of thyroid function hormone (TSH, FT3, and FT4) variables [14]. This sample (127 women) would have an 80% power and a difference of 5% at *α* = 0.05.

### Statistical analysis

All statistical analyses were performed with Statistical Product and Service Solutions (SPSS) software for Windows, version 22.0 (IBM, Armonk, New York, USA). Continuous data (including FT3, FT4, and TSH) were checked for normality using the Shapiro–Wilk test. The clinical and biochemical data for the independent variable _status of ID _and its two groups (women with ID and women without ID) were compared using either independent sample t- test for normally distributed data or Mann–Whitney U-Test for non-normally distributed data. Proportions were compared between two groups by Chi square test. Spearman correlations were performed between the continuous variables, and p-values of < 0.05 were considered significant.

## Results

### Characteristics of the women

In total, 127 pregnant women with median (IQR) age of 27.0 (23.0‒31.2) years participated in this study. The median (IQR) of the parity, gestational age, and BMI was 1 (1‒3), 10.0 (8.0‒13.0) weeks, and 26.6 (24.0‒29.7) kg/m^2^, respectively. The median (IQR) of TSH, FT3, and T4 was 1.600 (1.162‒2.092) IU/ml, 2.020 (1.772‒2.240) pmol/l, and 1.070 (0.960‒1.190) pmol/l, respectively (see Table 1).

**Table 1:** General characteristics of Sudanese women in early pregnancy (number, 127), 2021

Variables	
**Mean (standard deviation)**	
Age, years	27.0(5.5)
Gestational age, weeks	10.5(3.0)
**Median (Interquartile range) for**	
Parity	1(1–3)
Body mass index, kg/m^2^	26.6 (24.0 ‒29.7)
Hemoglobin, g/dl	10.9 (10.1‒11.7)
Serum ferritin, µg/l	19.4 (9.7‒33.7)
Thyroid-stimulating hormone, IU/ml	1.600 (1.162‒2.092)
Free triiodothyronine, pmol/l	2.020(1.772‒2.240)
Free thyroxin, pmol	1.070(0.960‒1.190)

Forty-seven (37.0%) of these 127 women had ID. The age, parity, gestational age, BMI, TSH, and FT3 were not different between women with ID and women without ID. The mean (SD) of FT4 was significantly lower in women with ID compared with women without ID [1.0 (0.17) vs. 1.11 (0.19) pmol, P = 0.007)] (see Table 2). Serum ferritin was inversely correlated with FT3 (r = -0.225, P = 0.011). There was no correlation between S ferritin, TSH, and FT4 (see Table 3).

**Table 2:** Comparing the clinical and biochemical characteristics of Sudanese women with iron deficiency and without iron deficiency, 2021

Variables	Women with iron deficiency (number = 47)	Women without iron deficiency(number = 80)	*P*
**Mean (standard deviation)** ‡			
Age, years	27.0(5.3)	28.2(5.7)	0.229
Gestational age, weeks	9.9(3.0)	10.9(2.9)	0.093
**Median (Interquartile range)** †			
Parity	1 (1‒3)	1 (1‒3)	0.514
Body mass index, kg/m^2^	27.1 (23.2 ‒30.8)	26.5 (24.3 ‒29.5)	0.576
Thyroid-stimulating hormone, IU/ml	1.628 (1.162‒2.010)	1.600 (1.147‒2.224)	0.485
Free triiodothyronine, pmol/l	2.060 (1.880‒2.310)	1.970 (1.762‒2.285)	0.086
Free thyroxin, pmol	1.020 (0.910‒1.120)	1.095 (0.990‒1.217)	0.014

‡ Independent sample t-test

† Mann Whitney *U* test

**Table 3:** Correlation between serum ferritin, thyroid functions, maternal age and gestational age

Variable	Serum ferritin		Thyroid-stimulating hormone		Free triiodothyronine		Maternal age		Gestational age	
	*r*	p	*r*	p	*r*	p	**r**	p	**r**	p
Free thyroxin	0.173	0.052	-0.077	0.387	0.003	0.973	-0.095	0.286	0.168	0.069
Serum ferritin			0.080	0.368	-0.225	0.011*	0.077	0.392	0.158	0.068
Thyroid-stimulating hormone					0.131	0.143	-0.117	0.189	0.044	0.627
Free triiodothyronine							-0.095	0.286	0.147	0.099
Maternal age									0.004	0.967

The values of FT3 and FT4 were within normal range for Sudanese pregnant women [15]. However, 31(24.4%) women had a higher TSH. Compared with women who had no ID, significantly, fewer patients with ID had high level of TSH (6/47[12.8%) vs.25/80 [31.3], *P* = 0.015).

## Discussion

This study investigated ID among pregnant women during the first trimester of the pregnancy. The prevalence of ID among pregnant women in this study was 37.0%, which is common given 41.9% was reported by a recent study that investigated the prevalence of ID among pregnant women in Sri Lanka [16].

The main findings of our study were that FT4 was significantly lower in women (in early pregnancy) with ID and that serum ferritin was inversely correlated with FT3. As mentioned above, different studies on ID and thyroid functions have shown different results; for example, He et al. reported that in women in the second trimester, the serum ferritin levels were negatively correlated with serum TSH levels and positively correlated with FT4 levels [8]. Hamed et al. reported a positive correlation between T3 and T4 and serum ferritin among 74 pregnant women in their early pregnancy [9]. Moreover, it has been reported that FT4 level was significantly lower and TSH value was significantly higher in women with ID (39.06% of them had ID, with serum ferritin < 15 µg/L) in the first trimester [3]. Likewise, in a large hospital-based study **(**168 cases with ID and 1,831 women without ID), FT3 and FT4 were significantly lower and TSH was significantly higher in pregnant women. In the latter study, authors reported that serum ferritin was positively correlated with FT3 and FT4 and inversely correlated with TSH [10]. In a recent study that was conducted on women in their first trimester of pregnancy, serum ferritin was a significant predictor of FT4 and T4 I [11]. The difference in the prevalence of ID between our study and others could explain the difference in these results. Our results showed that 37.0% of women had ID, while in He et al. study it was reported that only 11.4% of women had ID [8]. Further, while we used serum ferritin of less than 15 µg/L as the cut-off for ID, in the other study, authors used serum ferritin less than 12 µg/L as the cut-off for ID [8]. Likewise, the causes of ID could be different in different settings. For instance, in our setting, parasitic infection such as malaria was reported as the main cause of anemia during pregnancy [13]. It is noteworthy that in a recent meta-analysis (2021), pregnant women with ID had significantly increased serum TSH levels, decreased FT4 levels, and increased hypothyroidism [17].

Interestingly, in a large study that was conducted in India (491 women in early pregnancy), Savitha et al. reported no association between hypothyroidism and ID [12]. Iron has an important role in the function of the thyroid. For example, thyroid peroxidase (TPO), a heme-dependent protein, facilitates the actions of iodine in the thyroid [1]. Iron deficiency anemia is related to hypothyroidism [7]. Many studies have shown that ID can reduce or impair thyroid hormone synthesis and metabolism, possible through impairing the heme-dependent thyroid peroxidase (TPO) enzyme activity [3, 4, 7, 18]. Likewise, ID could lower TPO activity and thereby interfere with iodine metabolism in the thyroid. Thyroid peroxidase catalyzes the first two steps of thyroid hormone synthesis, iodination of thyroglobulin, and coupling of the iodo-tyrosine residues [18]. In addition, IDA can lead to a reduction of the peripheral conversion or even de-iodination of T4 to T3 and also change the central nervous system control of thyroid metabolism and nuclear T3 binding. Moreover, ID might impair through thyroid metabolism anemia and lowered oxygen transport [18]. Iron deficiency and thyroid abnormalities could have shared a common cause such as chronic inflammation and malnutrition [19]. It can also influence central nervous system functions and thus disturb the thyroid axis, which could influence thyroid hormone level [1]. Iron homeostasis is necessary for several biological processes, proteins, DNA, which are included in the thyroid functions [20].

This study has some limitations; first, the causes of ID such as inflammatory markers were not investigated in the current study. Second, due to fund constraints, thyroid antibodies were also not investigated.

## Conclusion

Iron deficiency was common during the first trimester of pregnancy and was associated with thyroid dysfunctions. Therefore, ID should be evaluated to avoid thyroid dysfunction.

## Data Availability

The data sets used or analyzed, or both during the current study are available from the corresponding author upon a reasonable request.

## List of Abbreviations

BMI: Body mass index
ID: Iron deficiency
IDA: Iron deficiency anemia
IQR: Interquartile range
FT3: Free triiodothyronine
FT4: Free thyroxine
TSH: Thyroid-stimulating hormone
